# Resveratrol Antagonizes Antimicrobial Lethality and Stimulates Recovery of Bacterial Mutants

**DOI:** 10.1371/journal.pone.0153023

**Published:** 2016-04-05

**Authors:** Yuanli Liu, Jinan Zhou, Yilin Qu, Xinguang Yang, Guojing Shi, Xiuhong Wang, Yuzhi Hong, Karl Drlica, Xilin Zhao

**Affiliations:** 1 Department of Biochemistry and Molecular Biology, Heilongjiang Provincial Science and Technology Innovation Team in Higher Education Institutes for Infection and Immunity, Harbin Medical University, 194 Xuefu Road, Nangang District, Harbin, Heilongjiang Province, 150081, China; 2 Public Health Research Institute and Department of Microbiology, Biochemistry, and Molecular Genetics, New Jersey Medical School, Rutgers, The State University of New Jersey, 225 Warren St, Newark, NJ 07103, United States of America; 3 State Key Laboratory of Molecular Vaccinology and Molecular Diagnostics, School of Public Health, Xiamen University, South Xiang-An Road, Xiang-An District, Xiamen, Fujian Province, 361102, China; University Paris South, FRANCE

## Abstract

Reactive oxygen species (ROS; superoxide, peroxide, and hydroxyl radical) are thought to contribute to the rapid bactericidal activity of diverse antimicrobial agents. The possibility has been raised that consumption of antioxidants in food may interfere with the lethal action of antimicrobials. Whether nutritional supplements containing antioxidant activity are also likely to interfere with antimicrobial lethality is unknown. To examine this possibility, resveratrol, a popular antioxidant dietary supplement, was added to cultures of *Escherichia coli* and *Staphylococcus aureus* that were then treated with antimicrobial and assayed for bacterial survival and the recovery of mutants resistant to an unrelated antimicrobial, rifampicin. Resveratrol, at concentrations likely to be present during human consumption, caused a 2- to 3-fold reduction in killing during a 2-hr treatment with moxifloxacin or kanamycin. At higher, but still subinhibitory concentrations, resveratrol reduced antimicrobial lethality by more than 3 orders of magnitude. Resveratrol also reduced the increase in reactive oxygen species (ROS) characteristic of treatment with quinolone (oxolinic acid). These data support the general idea that the lethal activity of some antimicrobials involves ROS. Surprisingly, subinhibitory concentrations of resveratrol promoted (2- to 6-fold) the recovery of rifampicin-resistant mutants arising from the action of ciprofloxacin, kanamycin, or daptomycin. This result is consistent with resveratrol reducing ROS to sublethal levels that are still mutagenic, while the absence of resveratrol allows ROS levels to high enough to kill mutagenized cells. Suppression of antimicrobial lethality and promotion of mutant recovery by resveratrol suggests that the antioxidant may contribute to the emergence of resistance to several antimicrobials, especially if new derivatives and/or formulations of resveratrol markedly increase bioavailability.

## Introduction

The increasing prevalence of antimicrobial resistance among bacterial pathogens has led to several approaches for addressing the problem. One is to develop new agents to replace old compounds whose efficacy has been eroded by resistance. Unfortunately, the most obvious antimicrobial targets have been identified, and derivatives of highly active antimicrobials have been extensively explored. Consequently, finding new agents is becoming increasingly difficult. Even big-data omics-based strategies have failed to meet expectations, as they have not produced a new antimicrobial despite of a decade of effort [1]. Another approach, restricting use, has shown some success [2–5], but it is clear that restricting consumption will not solve the problem [3,6]. A third strategy is to raise doses to block mutant amplification [7]. This approach is restricted by potentially adverse effects from elevated doses. We have taken a fourth approach by seeking ways to make existing agents more lethal [8,9]: rapid killing of bacteria should suppress the effects of mutagenic stress responses, such as induction of the SOS regulon.

Recent work on antibacterial lethality has focused on the proposal by Kohanski *et al*. [10] that reactive oxygen species (ROS) contribute to killing. Antimicrobials of various types are thought to create distinctive lesions that block growth and, if sufficiently severe, prompt a lethal stress response that culminates in a cascade of toxic ROS [11,12]. Several types of evidence support this idea. For example, agents and genes thought to interfere with ROS accumulation also interfere with the lethal action of diverse antimicrobials [10,13,14], and members of genetic pathways leading to a surge in ROS contribute to antimicrobial lethality [10,15–19]. Moreover, the end stage, cell death, has been attributed largely to double-strand DNA breaks arising from ROS-mediated formation of 8-oxo-guanidine [20]. Thus, ROS appear to contribute to rapid killing by diverse agents (fluoroquinolones, aminoglycosides, β-lactams, and polymyxins). We emphasize the term “rapid killing” because long incubation periods with antimicrobial, as is common with the measurement of minimal bactericidal concentration (MBC), fail to reflect the accelerating effect ROS have on killing [14,21].

The accumulation of ROS may not fully account for antimicrobial lethality. For example, with some antimicrobials the lesions themselves are sufficient to kill cells, thereby adding to the ROS effect or even pre-empting it [22]. Indeed, the ROS-lethality hypothesis has been challenged [23,24]. The challenges have been rebutted [13], and issues involving ROS have been clarified [11,12,25]. Nevertheless, additional tests of the hypothesis continue to be important.

Among the tests for ROS involvement in antimicrobial lethality are measurements of antioxidant-mediated interference of killing by antimicrobials. For example, thiourea, glutathione, and vitamin C reduce the lethal activity of fluoroquinolones by orders of magnitude [10,14,21,26]; thiourea and glutathione reduce lethality of daptomycin and oxacillin by 10–100 fold [21]. Antioxidant effects may also extend to their consumption by humans. For example, Marathe *et al*. [27] examined effects of the antioxidant curcumin on ciprofloxacin-mediated lethality with *Salmonella*. Curcumin, which is a common food ingredient in Southeast Asia and is often used medicinally, reduces the lethal activity of ciprofloxacin with cultured bacteria, in macrophage-like cells infected with bacteria, and in a mouse infection model. Since curcumin also suppresses the antibacterial activity of the immune response, it potentially acts in two ways to increase bacterial survival [27]. Thus, a cautionary note has been raised concerning antibiotic therapy and consumption of foods having antioxidant activity [27].

Another potential test for ROS involvement in antimicrobial lethality involves antioxidant nutritional supplements. One of the popular compounds is resveratrol, a natural polyphenol antioxidant [28] thought to have beneficial effects for ailments such as cardiovascular disease [29], neurodegenerative disorders [29,30], and some forms of cancer [29,31]. In the present work we found that resveratrol interferes with the lethal action of several antimicrobials with two bacterial species, *Escherichia coli* and *Staphylococcus aureus*. While the effects were small at resveratrol concentrations commonly found in human serum, those at higher concentrations clearly support the ROS-antimicrobial lethality hypothesis and emphasize the need to evaluate the nutritional antioxidants for effects on antimicrobial chemotherapy.

## Materials and Methods

### Bacterial strains and reagents

Liquid cultures of *E*. *coli* strain BW25113 and *S*. *aureus* strain RN450 were grown in LB or Muller-Hinton broth (BD-Difco, Franklin Lakes, NJ), respectively, at 37°C. At mid-exponential phase, the cultures were treated with a variety of antimicrobials in the presence or absence of resveratrol at various concentrations.

Resveratrol and other reagents, including antimicrobials, were purchased from Sigma-Aldrich (St. Louis, MO). Exceptions were moxifloxacin and ciprofloxacin, which were obtained from Bayer AG (Wuppertal, Germany), and daptomycin (Cubist Pharmaceuticals, Lexington, MA). Carboxy-H_2_-DCFDA was purchased from Invitrogen (Carlsbad, CA)

### Measurement of antibacterial susceptibility and mutant recovery

Minimal inhibitory concentration (MIC) was determined by broth dilution using 2-fold increments of antimicrobial with bacterial aliquots containing approximately 10^5^ cfu/ml. The lowest drug concentration that inhibited visible overnight growth was taken as MIC. Minimal bactericidal concentration (MBC) was determined as for MIC except that larger inocula (10^6^ to 10^7^ cfu/ml) were used and bacterial survival was assessed by plating post-treatment samples on drug-free agar. The lowest antimicrobial concentration that reduced viability by 99.9% was taken as MBC.

To determine rapid lethal activity, exponentially growing bacterial cultures (~5 x 10^8^ cfu/ml) were incubated with antimicrobial in the presence or absence of a sub-inhibitory concentration of resveratrol. After incubation, cultures were diluted in 0.9% sterile saline, plated on drug-free agar, and incubated overnight at 37°C to determine percent survival relative to an untreated control obtained at the time of antimicrobial addition.

Mutant recovery was measured by plating antimicrobial/resveratrol-treated cultures on agar containing the unrelated antibiotic rifampicin (5 x MIC) and scoring rifampicin-resistant colonies appearing every 24 hr for 72 hr. An apparent mutation frequency was calculated by dividing the number of colonies recovered on rifampicin-containing agar by that recovered on drug-free agar.

### Measurement of reactive oxygen species

Intracellular accumulation of ROS was measured by fluorescence-based flow cytometry using carboxy-H_2_DC-FDA, a dye that becomes fluorescent upon reaction with ROS [32]. *E*. *coli* cells were grown to early exponential phase (~ 2.5 X 10^8^ cells/ml) and then treated with 10 μM carboxy-H_2_DCFDA for 20 min before cultures were administered oxolinic acid alone (20 X MIC, 8 μg/ml), resveratrol alone (0.5 X MIC, 200 μg/ml), or oxolinic acid plus resveratrol for an additional 120 min. Samples taken before and after oxolinic acid treatment were subjected to flow cytometry analysis using an Accuri C6 flow cytometer (BD Accuri Cytometers, Ann Arbor, MI). A total of 100,000 cells were counted for each sample.

### Statistical analysis

Statistical data analysis was performed using Student’s t-test. A *p* value <0.05 was considered significantly different.

## Results

### Resveratrol reduces rapid antimicrobial lethality with little effect on MIC or MBC

**Fig 1:**
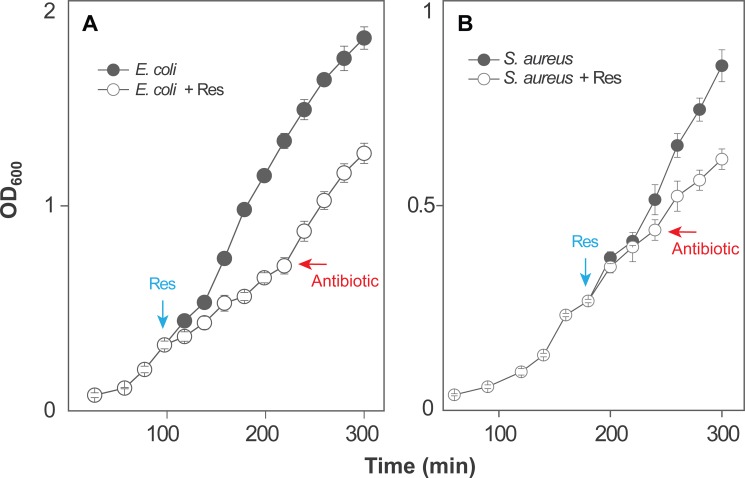
Effect of resveratrol on bacterial growth rate. Liquid cultures of *E*. *coli* (A) or *S*. *aureus* (B) were grown in the presence (empty circles) or absence (filled circles) of resveratrol (½ MIC). Culture turbidity was monitored as OD_600_. Down-pointing arrows labeled Res indicate time at which resveratrol was added. Left-pointing arrows labeled Antibiotic indicate the culture density at which antimicrobial was added. The experiments were performed at least 3 times with similar results; error bars indicate standard error of means from triplicate plating of a representative experiment.

**Fig 2:**
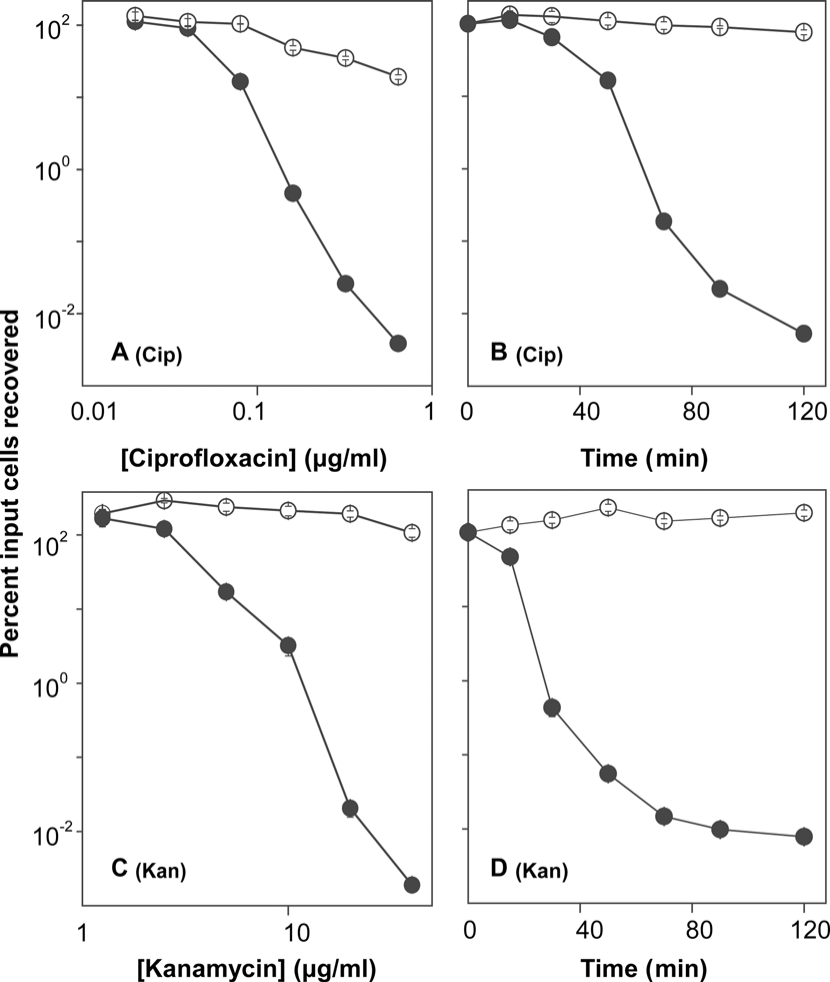
Effect of resveratrol on ciprofloxacin and kanamycin lethality with *E*. *coli*. Exponentially growing *E*. *coli* cultures were incubated with various concentrations of antimicrobial for a fixed time (A, C) or with a fixed concentration of antimicrobial for various times (B, D) in the presence (empty circles) or absence (filled circles) of resveratrol at a subinhibitory concentration (0.2 mg/ml; ½ MIC). After incubation, cultures were diluted in 0.9% sterile saline, plated on drug-free agar, and incubated overnight at 37°C to determine percent survival relative to an untreated control obtained at the time of antimicrobial addition. Panel A: ciprofloxacin (Cip) at the indicated concentrations incubated for 45 min. Panel B: ciprofloxacin incubated for the indicated times at 5 x MIC. Panel C: kanamycin (Kan) incubated at the indicated concentrations for 30 min. Panel D: kanamycin incubated for the indicated times at 10 x MIC. Each experiment was performed at least 3 times with similar results; error bars indicate standard error of means from triplicate plating of a representative experiment.

**Fig 3:**
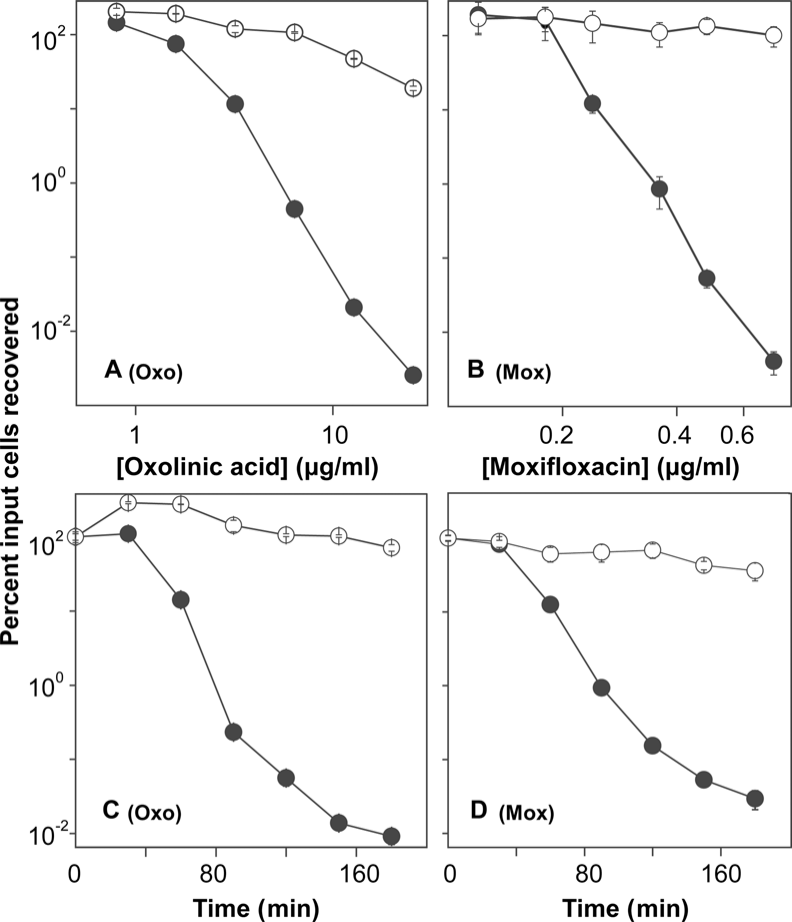
Resveratrol-mediated inhibition of rapid killing of *E*. *coli* by oxolinic acid and moxifloxacin. Panel A: Exponentially growing *E*. *coli* cultures were incubated with various concentrations of oxolinic acid (Oxo) for 2 hr. Panel B: Cultures were incubated with the indicated concentrations of moxifloxacin (Mox) for 1 hr. Panel C: Cultures were incubated for the indicated times with oxolinic acid at 20 x MIC. Panel D. Cultures were incubated for various times with moxifloxacin at 15 x MIC. Symbols: empty circles (presence of resveratrol at ½ MIC); filled circles (absence of resveratrol). After incubation, percent survival was determined as in Fig 2. Each experiment was performed at least 3 times with similar results; error bars indicate standard error of means from triplicate plating of a representative experiment.

**Fig 4:**
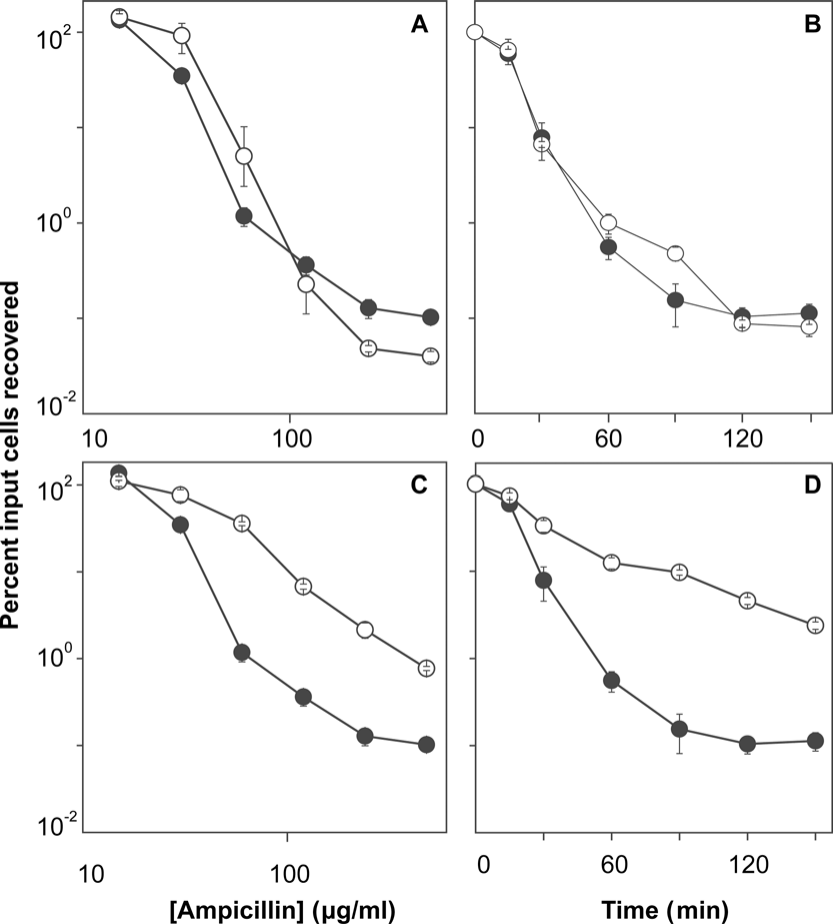
Effect of resveratrol on ampicillin-mediated killing of *E*. *coli*. Exponentially growing *E*. *coli* cultures were incubated with two different concentrations of resveratrol. Panel A. Cultures were incubated with the indicated concentrations of ampicillin for 2 hr at ½ MIC resveratrol. Panel B. Cultures were incubated with ampicillin for the indicated times at 10 x MIC with ½ MIC resveratrol. Panel C. Cultures were incubated with ampicillin at the indicated concentrations for 2 hrs with ¾ MIC resveratrol. Panel D. Cultures were incubated for the indicted times with ampicillin at 10 x MIC with ¾ MIC resveratrol. Symbols: empty circles (presence of resveratrol); filled circles (absence of resveratrol). After incubation, percent survival was determined as in Fig 2. Each experiment was performed at least 3 times with similar results; error bars indicate standard error of means from triplicate plating of a representative experiment.

**Fig 5:**
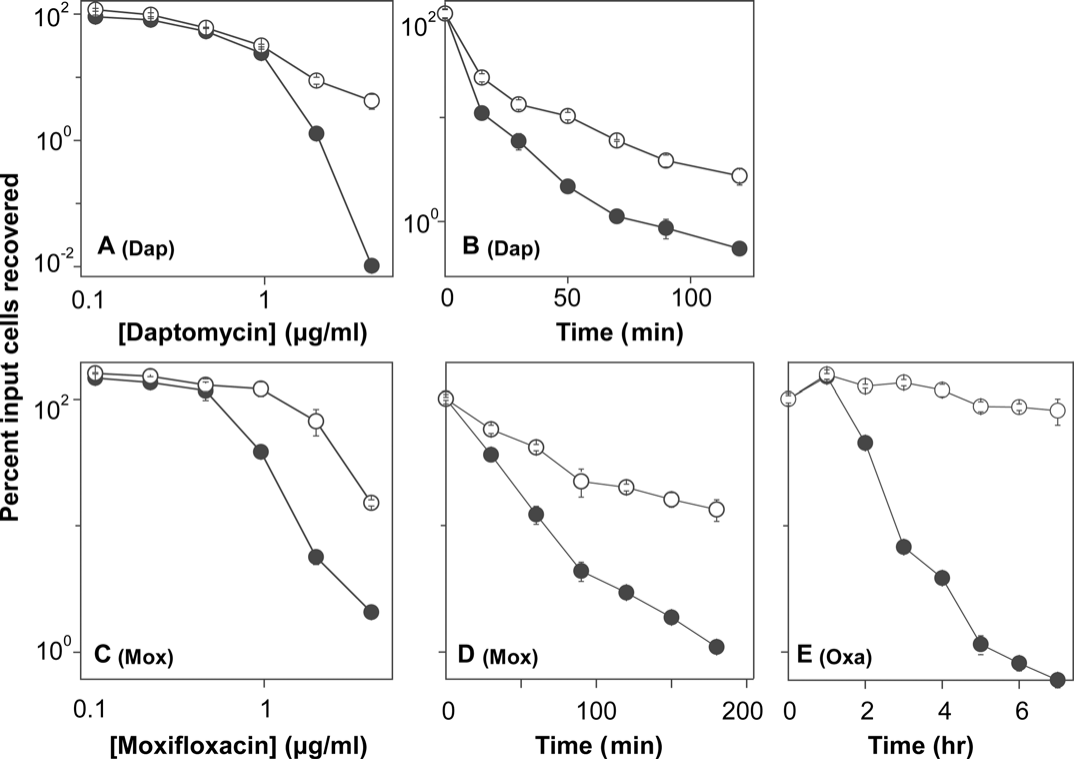
Effect of resveratrol on antimicrobial lethality with *S*. *aureus*. Exponentially growing cultures of *S*. *aureus* were treated with antimicrobials in the same manner as *E*. *coli* (Fig 2) except that different antimicrobials were tested as indicated and resveratrol concentration was at 75 μg/ml (1/2 MIC). Panel A. Effect of daptomycin (Dap) concentration with a 1-hr incubation. Panel B. Effect of incubation time with daptomycin at 32 x MIC. Panel C. Effect of moxifloxacin (Mox) concentration with a 2-hr incubation. Panel D. Effect of incubation time with moxifloxacin at 50 x MIC. Panel E. Effect of incubation time with oxacillin (Oxa) at 50 x MIC. Each experiment was performed at least 3 times with similar results; error bars indicate standard error of means from triplicate plating of a representative experiment.

To determine whether resveratrol protects bacteria from rapid antimicrobial-mediated killing, we first measured MIC (Table 1) to identify a sub-inhibitory concentration and thereby avoid resveratrol-mediated growth inhibition that is expected to interfere with killing by many antimicrobials. Resveratrol had an MIC of 400 and 150 μg/ml with *E*. *coli* and *S*. *aureus*, respectively, which is consistent with previous reports showing that resveratrol itself has antibacterial activity at high concentrations [33,34]. When ½ MIC of resveratrol was added to exponentially growing cultures, growth of *E*. *coli* was delayed for about 1 hr, but then it attained roughly the same rate as an untreated culture (Fig 1A). With *S*. *aureus*, resveratrol reduced growth rate by approximately 40% (Fig 1B). When added to growing cultures, resveratrol reduced rapid killing with *E*. *coli* for both ciprofloxacin and kanamycin by 100- to 1,000-fold when either antimicrobial concentration or incubation time was varied (Fig 2A–2D). A protective effect of resveratrol on killing was also observed with two other quinolones, oxolinic acid and moxifloxacin (Fig 3). The lethal effect of ampicillin was insensitive to ½ MIC resveratrol, but partial protection was observed at ¾ MIC (Fig 4). With *S*. *aureus*, resveratrol reduced the rapid lethality of daptomycin and moxifloxacin; killing by oxacillin was blocked (Fig 5). Thus, the rapid lethal action of several antimicrobial classes is reduced by resveratrol, with the magnitude of the effect varying among the compounds. These data show that resveratrol lowers antimicrobial-mediated lethality with both Gram-negative and Gram-positive bacteria. Further investigation is required to determine whether resveratrol-mediated effects apply to many bacterial species.

**Table 1:** Effect of resveratrol on minimal inhibitory and minimal bactericidal concentrations with *E*. *coli* and *S*. *aureus*.

Compound	MIC; MBC (μg/ml)	
	*E*. *coli* (BW25113)	*S*. *aureus* (RN450)
Resveratrol (Res)	400;NA^a^	150;NA^a^
Ciprofloxacin (Cip)	0.02;0.04	0.5;2.0
Cip + Res^b^	0.01;0.04	0.25;1.0
Moxifloxacin (Mox)	0.03;0.06	0.06;0.24
Mox + Res^b^	0.06;0.12	0.06;0.24
Ampicillin (Amp)	15;30	NA^a^
Amp + Res^b^	15;30	NA^a^
Oxacillin (Oxa)	NA^a^	0.25;0.5
Oxo + Res^b^	NA^a^	0.25;0.5
Kanamycin (Kan)	1.25;5.0	NA^a^
Kan + Res^b^	2.5;10	NA^a^
Oxolinic acid (Oxo)	0.4;0.8	NA^a^
Oxo + Res^b^	0.8;1.6	NA^a^
Daptomycin (Dap)	NA^a^	0.06;0.48
Dap + Res^b^	NA^a^	0.06;0.48
Rifampicin	8;NA^a^	0.0075;NA^a^

^a^Not applicable/available.

^b^Half MIC of resveratrol was included in growth medium for determination of MIC and MBC.

**Fig 6:**
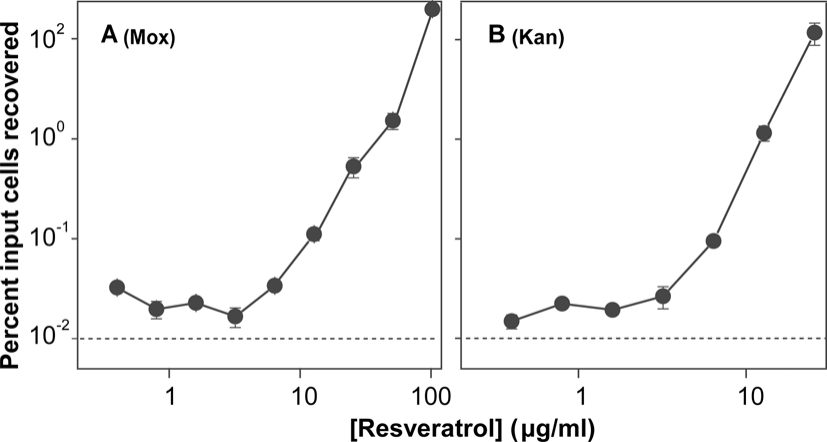
Effect of resveratrol concentration on lethality of moxifloxacin and kanamycin with *E*. *coli*. Exponentially growing cultures of *E*. *coli* were incubated with moxifloxacin (Mox, 8 x MIC, panel A) or kanamycin (Kan, 3 x MIC, panel B) for 2 hr in the presence of various concentrations of resveratrol. After incubation, bacterial survival was determined as in Fig 2 and plotted as a function of resveratrol concentration. Survival in the absence of resveratrol for moxifloxacin and kanamycin, under the same conditions, was 0.01% (indicated as dashed lines in the figure, since zero concentration of resveratrol cannot be plotted in a log scale). Each experiment was performed at least 3 times with similar results; error bars indicate standard error of means from triplicate plating of a representative experiment.

While the resveratrol concentration in the experiments described above was below MIC, the concentration was far above human serum concentrations attained following ingestion of typical doses (resveratrol plasma levels can reach 1 μg/ml with a single dose of 5 g [35]). To determine whether resveratrol-mediated interference of antimicrobial lethality is concentration dependent, we examined a broad range of resveratrol concentration for protection of *E*. *coli* from rapid killing by moxifloxacin and by kanamycin. A striking concentration dependence was observed (Fig 6), with resveratrol increasing bacterial survival by as much as 1000-fold at concentrations that were subinhibitory for resveratrol. Even at the low resveratrol concentrations found in human serum after safe mega doses, bacterial survival was increased by 2–3 fold. Determining whether this low level of protection is clinically significant requires additional work that will include understanding how daily use by very large numbers of persons affects the emergence of resistance (see below).

It has been argued that the major effects of ROS result from a cellular response to lesions introduced by antimicrobials [11,12]. Those lesions block bacterial growth, but, depending on antimicrobial concentration, the lesions may or may not elicit a lethal cascade of ROS. In the present work, resveratrol caused at most a 2-fold increase in antimicrobial MIC, indicating that bacteriostatic antimicrobial concentrations have little stimulating effect on the accumulation of ROS. This result is consistent with previous studies using other antioxidants [10,14,21]. We have also argued that accumulation of ROS accelerates killing by antimicrobials but does not increase the extent [21]. As expected, resveratrol had no effect on MBC for several antimicrobials (Table 1): determination of MBC involves a long (overnight) incubation that would be insensitive to rate of killing.

### Resveratrol increases mutant recovery frequency

**Fig 7:**
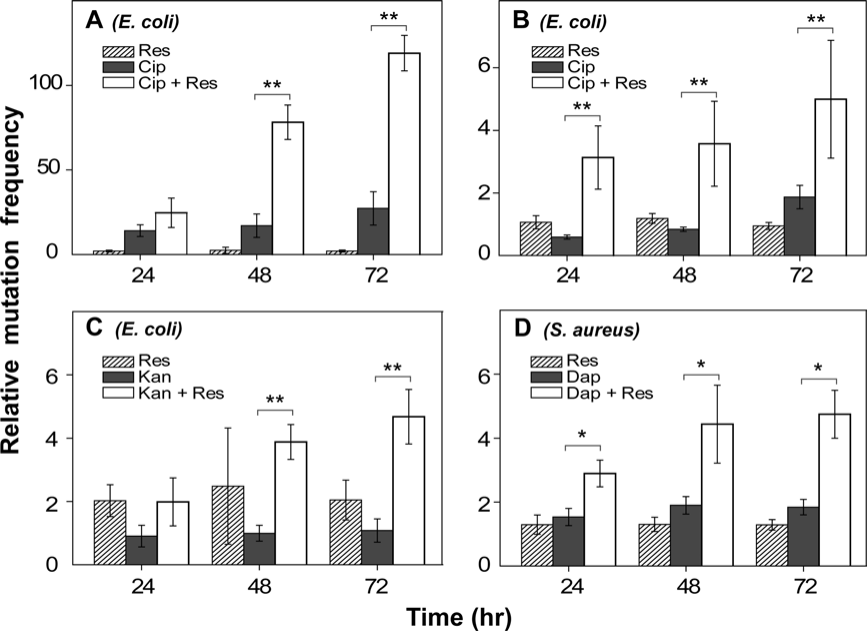
Effect of resveratrol on the recovery of antimicrobial-induced rifampicin-resistant mutants. Exponentially growing cultures of *E*. *coli* (A, B, C) or *S*. *aureus* (D) were pretreated with ciprofloxacin (Cip, 5 x MIC for 45 min in panel A; 3 X MIC for 1 hr in panel B), kanamycin (Kan, 2 x MIC for 30 min, panel C); cultures of *S*. *aureus* were treated with daptomycin (Dap, 15 x MIC for 60 min, panel D) in the presence (white bars) or absence (black bars) of resveratrol (½ MIC for A, C, D; 0.8 μg/ml for B). Untreated and resveratrol-only cultures (hatched bars) were also included as controls. After incubation, cultures were spread on agar containing 5 x MIC of rifampicin to assess mutation frequency following incubation at 37°C cfu determination. Apparent mutation frequency was calculated using colony numbers recovered on rifampicin-containing agar relative to colony numbers recovered on drug-free agar for each sample at the time of plating. Mutation frequency from the untreated culture was set at 1 while those from various treatments were expressed relative to the untreated control. All experiments were performed 3 times. Error bars indicate standard error of means. * indicates p<0.05, ** p < 0.01.

Another feature of antimicrobial-mediated accumulation of ROS is mutagenesis [36]. For example, 8-oxo-guanine, which is generated by ROS [20], is a well-known mutagen [37], and antimicrobial treatment is known to be mutagenic [38,39], in part through stimulation of ROS [36]. Thus, we expected resveratrol to dampen the recovery of mutants arising from antimicrobial treatment, especially since that is the case when resveratrol is used to inhibit genotoxicity by heterocyclic amines with both bacterial and mammalian cells [40]. As a test, we treated bacterial cultures with antimicrobial in the presence or absence of resveratrol, and then we plated cells on agar containing an unrelated antibiotic, rifampicin, to probe the apparent mutation frequency. With *E*. *coli*, ciprofloxacin, when alone, raised the recovery of rifampicin-resistant mutants by more than 20-fold (Fig 7A). Resveratrol alone showed little (< 2-fold) effect on mutant recovery frequency. Addition of resveratrol (½ MIC) to ciprofloxacin raised the recovery of mutants by another 6-fold beyond the effect of ciprofloxacin alone (Fig 7A). Even at low, serum-achievable concentrations (e.g. 0.8 μg/ml), resveratrol stimulated the frequency of ciprofloxacin-induced resistance to rifampicin by 2–3 fold (Fig 7B). With kanamycin alone, no mutagenic action was detectable (Fig 7C), but the additional presence of ½ MIC resveratrol increased the recovery of kanamycin-induced rifampicin-resistant colonies by 2- to 4-fold (Fig 7C). A similar phenomenon was observed for daptomycin with *S*. *aureus* (Fig 7D). Collectively these data indicate that resveratrol promotes the recovery of mutants, which could, in principle, contribute to the emergence of antibiotic resistance.

### Resveratrol decreases ROS levels stimulated by oxolinic acid

**Fig 8:**
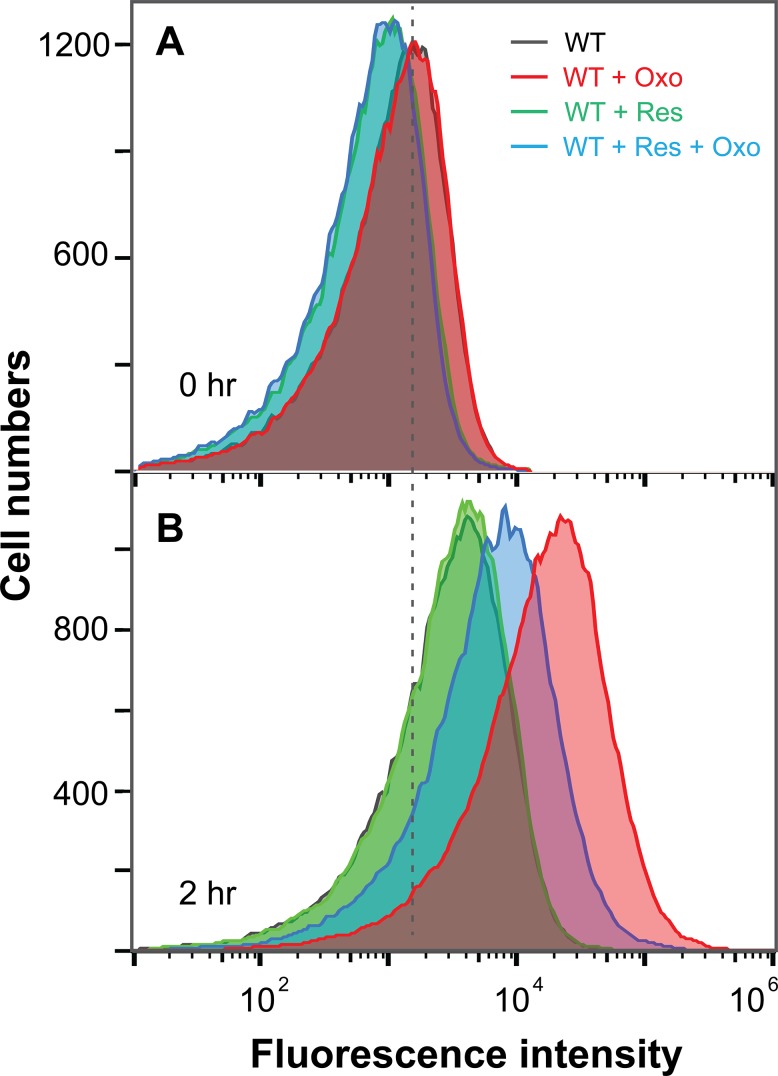
Accumulation of reactive oxygen species in *E*. *coli* treated with oxolinic acid with or without resveratrol. Exponentially growing *E*. *coli* cultures (~ 2.5 X 10^8^ cells/ml) were treated with 10 μM carboxy-H_2_DCFDA for 20 min before cells were administered oxolinic acid alone (WT + Oxo; 20 X MIC, 8 μg/ml), resveratrol alone (WT + Res; 0.5 X MIC, 200 μg/ml), or oxolinic acid plus resveratrol (WT + Res + Oxo) for an additional 120 min. Samples taken before (A) and after (B) oxolinic acid treatment were subjected to flow cytometry analysis. Similar results were observed with 3 replicate experiments. Legend: Control samples (WT), black lines; resveratrol alone (WT + Res), green lines; oxolinic acid alone (WT + Oxo), red lines; and resveratrol plus oxolinic acid (WT+ Res + Oxo), blue lines.

We examined *E*. *coli* cultures treated with oxolinic acid to determine whether resveratrol suppresses antimicrobial-stimulated accumulation of ROS (we chose oxolinic acid for this experiment because previous work showed that this quinolone stimulates measureable levels of ROS [16] and because the lethal activity of oxolinic acid depends largely on ROS accumulation [22]). For this experiment, we pretreated *E*. *coli* for 20 min with carboxyl-H_2_DC-FDA, a compound that can penetrate cell membranes but cannot leak out once intracellular esterases convert it into a membrane non-permeable form [32,41]. Reaction with ROS makes the compound fluorescent, and fluorescence is measured by flow cytometry [32]. Oxolinic acid (8 μg/ml, 20 X MIC) was then added, and after 120 min cells were harvested and examined by flow cytometry. As shown in Fig 8, oxolinic acid alone shifted the curve, showing increased fluorescence intensity; resveratrol reduced the shift. We conclude that resveratrol restricts quinolone-mediated accumulation of intracellular ROS.

## Discussion

The work described above showed that at subinhibitory concentrations (½ MIC) resveratrol, an antioxidant [28] widely used as a nutritional supplement, interferes with the lethal action of ciprofloxacin, oxolinic acid, moxifloxacin, and kanamycin with *E*. *coli* (Figs 2 and 3); the lethal action of ampicillin was reduced at ¾ MIC resveratrol (Fig 4). With *S*. *aureus*, resveratrol protected from the lethal action of daptomycin, moxifloxacin, and oxacillin (Fig 5). The magnitude of the protective effect varied among the test compounds; in some cases it was almost complete, while in others it was only partial. As expected for an antioxidant, resveratrol interfered with the accumulation of ROS stimulated by the quinolone oxolinic acid (Fig 8). We note that resveratrol had little effect on antimicrobial MIC (Table 1), which emphasizes that the effects of ROS are largely on the lethal rather than the bacteriostatic properties of antimicrobials [12]. It also had little effect on MBC (Table 1), which reinforces the point that ROS accelerate the lethal activity of antimicrobials. Overall, these data support the hypothesis that ROS contribute to the lethal activity of diverse antimicrobials [10,11,25].

Resveratrol also increased the recovery of antibiotic-induced mutants that were monitored as resistance to an unrelated antibiotic (Fig 7). This result was unexpected, because antibiotic-induced ROS lead to production of 8-oxo-guanine [20], which is mutagenic [37]. Since sublethal concentrations of antimicrobials (norfloxacin, ampicillin, kanamycin) can be mutagenic, probably via ROS accumulation [36], the suppression of ROS by resveratrol appears to eliminate/suppress bacterial killing but leaves sufficient residual ROS to generate mutations. Thus, antioxidants may contribute in two ways to the emergence of antimicrobial resistance: reduction of lethal activity and increased recovery of mutants.

Although the protective effect of resveratrol on antimicrobial-mediated killing is small at concentrations found in the serum of persons currently consuming the compound (*E*. *coli* survival increase of 2–3 fold for moxifloxacin and kanamycin), efforts are being made to increase the bioavailability of resveratrol [42,43]. Moreover, resveratrol concentrations in tissues can be higher than in serum [44], and resveratrol metabolites, some of which show radical-scavenging activity similar to that of resveratrol [45], have higher bioavailability than resveratrol and can serve as an intracellular reservoir from which parental resveratrol can be regenerated [44]. Furthermore, some derivatives of resveratrol, such as pterostilbene, can achieve a 10-fold higher peak plasma concentration [46]. Thus, the effects we report may be important, especially since large numbers of persons consume resveratrol and other antioxidants (half of the U.S. population consumes dietary supplements [47], most of which contain antioxidants).

In summary, the nutritional antioxidant work presented above supports the general idea that the accumulation of toxic ROS contributes to the lethality of diverse antimicrobials [10]. Moreover, the work fits well with an earlier study of curcumin, a common ingredient in Indian diets [48], that interferes with the lethal activity of ciprofloxacin [27]. The present report extends the antioxidant concept to another Gram-negative species (*E*. *coli*), to a Gram-positive species (*S*. *aureus*), to other lethal antimicrobials (kanamycin, daptomycin, and oxacillin), and to dietary supplements commonly used in Western societies.
